# Effects of hydroalcoholic extract of *Solidago chilensis* Meyen on nociception and hypernociception in rodents

**DOI:** 10.1186/s12906-019-2478-8

**Published:** 2019-03-20

**Authors:** Elena L. A. Malpezzi-Marinho, Graziela R. Molska, Lyvia I. G. P. Freire, Cristiane I. Silva, Eduardo K. Tamura, Laís F. Berro, Carlos A. Parada, Eduardo Ary Villela Marinho

**Affiliations:** 1 Department of Biological Sciences, Universidade Estadual de Santa Cruz, BR-415, Rodovia Ilhéus- Itabuna, Km-16, Salobrinho, Ilhéus, Bahia 45662-000 Brazil; 2 Faculdade de Ciências da Saúde, Universidade Braz Cubas, São Paulo, Brazil; 3 Department of Pharmacology, Faculdade de Ciências Médicas, Universidade de São Paulo, Ribeirão Preto, SP Brazil; 4 Department of Psychiatry and Human Behavior, University of Mississippi Medical Center, Jackson, MS USA; 5 Institute of Biology, Universidade Estadual de Campinas, Campinas, SP Brazil; 6 Department of Health Sciences, Universidade Estadual de Santa Cruz, BR-415, Rodovia Ilhéus- Itabuna, Km-16, Salobrinho, Ilhéus, Bahia 45662-000 Brazil

**Keywords:** *Solidago chilensis* Meyen, Pain, Nociception, Motor activity, Rats, Mice

## Abstract

**Background:**

*Solidago chilensis* (syn. *microglossa*) is a plant from the Asteraceae family widely distributed in South America and used to treat inflammatory diseases. In 2009, it was listed as one of the native medicinal herbal plants used in the Brazilian public health system. In addition to its anti-inflammatory properties, a recent clinical study has shown antinociceptive effects of *S. chilensis*, introducing a new potential medical use for this plant. The aim of the present study was to investigate the antinociceptive activity of the hydroalcoholic extract of *Solidago chilensis* (HESc) in rodent models of pain.

**Methods:**

The dried plant extract was obtained from its aerial parts, maintained in ethanol (100 g/l) and filtered. Rats or mice were treated with intraperitoneal injections of HESc (3, 10 or 30 mg/kg) 30 min before being submitted to writhing, 0.2%-formaline or hot-plate tests or prostaglandin E_2_ (PGE_2_) administration in the hind paw. Mechanical hypernociception and motor impairment were evaluated by electronic von Frey and rota-rod, respectively.

**Results:**

HESc dose-dependently inhibited abdominal contortions in the writhing test and attenuated phases I and II formalin-induced nociceptive behavior. Treatment with HESc also increased thermal threshold and decreased PGE_2_-induced hypernociception without promoting motor impairment.

**Conclusions:**

Our data suggest that, when systemically administered, HESc decreases nociception without inducing a sedative effect. Importantly, this effect was observed in both inflammatory and non-inflammatory models of pain and nociception, suggesting a specific non-inflammatory mechanism of HESc on pain. Our findings indicate that *S. chilensis* might be an important adjuvant in pain management.

## Background

The genus *Solidago* is considered one of the largest genera of the Asteraceae family of plants, comprising more than 120 species, most of which are found in North and South America [1]. This genus comprises a great number of medicinal plants with therapeutic properties. Among them, *S. chilensis* Meyer (syn. *microglossa*) has been recently considered for the treatment of inflammatory diseases. Studies have demonstrated that aqueous or hydroalcoholic extracts of *S. chilensis* or its essential oil contain compounds with relevant anti-inflammatory activity similar to those of dexamethasone [2–4].

In Brazil, where the plant is wildly found, the extract of *S. chilensis* is currently indicated as a therapeutic tool for contusion, trauma and boils by Medicinal Plant Programs [5]. Since 2009, *S. microglossa* was included in the national list of native medicinal plants used by the Brazilian public health system (SUS). *S. chilensis* is used topically or by oral administration, depending on the region and according to cultural traditions. Patches from leaves and stems are used for muscle pain or skin lesions [6, 7]. Infusion, bath, decoction or tincture of *S. chilensis* flowers or the whole plant macerated are also used for wound healings and boils, purulent infections, inflammation, edema, body pain, chill or rheumatism [8–10].

In light of its traditional medical use, pre-clinical studies in rodents have investigated the therapeutic effects of *S. chilensis* for a variety of conditions, with encouraging results. *S. chilensis* has been shown to exert antifungal activity in in vitro assays [11, 12]. The plant also induces gastroprotective activity in rodents [13, 14], and ameliorates insulin resistance in obese mice [15]. Of note, previous studies in rodents have also shown that *S. chilensis* exerts an anti-inflammatory modulatory role in murine models of pleurisy [2], air pouch [3], and ear oedema [4]. Based on its anti-inflammatory properties in animal models and its traditional medical use, clinical trials have recently investigated the potential analgesic effects of *S. chilensis* extract in humans. Volunteers with lumbago [16] and tendinitis of flexor and extensor tendons of wrist and hand [17] were locally treated with topical gel containing *S. chilensis* extract. In both studies, patients reported significant reduction in the perception of pain compared to placebo. Although those studies included small sample sizes (20 and 8 participants, respectively), they introduce a new field of investigation on *S. chilensis* that might have important clinical implications for pain management.

The aim of the present study was to verify whether the extract of *S. chilensis* exerts antinociceptive and anti-hypernociceptive effects in different experimental models in rodents. Because of the proven anti-inflammatory action of this plant, we investigated the effects of a hydroalcoholic extract of *S. chilensis* in models of inflammatory pain, including the writhing test and the formalin test. In order to test if *S. chilensis* also exerts significant effects on the central transmission of pain, we investigated the effects of its extract in models of non-inflammatory pain, such as nociceptive response to heat or a mechanical stimulus.

## Methods

### Botanical material

*Solidago chilensis* Meyen (plant name checked with http://www.theplantlist.org) was collected in the city of Guararema, São Paulo, Brazil. Botanical identification and authentication were performed by Dr. Lucia Rossi from the Instituto Botânico de São Paulo, São Paulo, Brazil, and one specimen was deposited in the herbarium under the number SP 397.047. The aerial parts of the plant (flowers, leaves and stalks) were used in the extract.

### Hydroalcoholic extract of Solidago chilensis (HESc)

The aerial parts of the plant were immersed in 93% ethanol (100 g/l) for one month. After this period, the hydroalcoholic extract was filtered, concentrated using a rotary evaporator and then lyophilized. The dried material was stored at 4 °C until use. The dried extract was diluted in saline immediately prior to use. Solutions were given intraperitoneally (i.p.) at a volume of 10 ml/kg of body weight.

The chemical characterization of the HESc employed in the present study has been described in a previous study from our group using the same extract [4]. Analysis via HPLC–DAD–MS and HPLC–MS/MS employing the analytical methodology developed by Gobbo-Neto and Lopes [18] demonstrated that chromatograms obtained for the hydroalcoholic extract presented 4 main peaks, that were identified as 5-O-E-, 3,4-, and 4,5-di-OE-caffeoylquinic acids and rutin.

### Animals

Male 3-month-old Swiss mice (25–35 g) and Wistar rats (200–250 g) were obtained from the vivarium of the University of São Paulo/Ribeirão Preto or the Braz Cubas University. Animals were group housed in polypropylene cages (5–7 per cage for mice; 3–4 per cage for rats) under controlled temperature (22 ± 1 °C) in a light/dark cycle of 12 h (lights on at 7:00 am) with free access to food (chow) and water. All protocols and animal care and handling strictly followed the National Institutes of Health Guide for the Care and Use of Laboratory Animals (8th edition, revised 2011), and animals were maintained in accordance with the Brazilian Law for Procedures for Animal Scientific Use (#11794/2008). All animals were naïve prior to the beginning of the study. At the end of the study, animals were submitted to 35% CO_2_ euthanasia. All experimental procedures were approved by the Institutional Ethical Committee from Braz Cubas University (protocol #175/2008) and University of São Paulo/Ribeirão Preto (protocol # 11794/2008).

### Experimental procedures

All experiments were performed during the same time of day (8 am-12 pm). The doses and route of administration (intraperitoneal) used were chosen based on previous studies from our group investigating the effects of HESc on acute inflammation [4]. In our previous study, only topical and intraperitoneal, but not oral, administration of the extract exerted anti-inflammatory effects in rat ear oedema, suggesting that the absorption of its anti-inflammatory component(s) may be affected by the gastrointestinal tract [4]. For all experiments described below, animals were randomly assigned to different experimental groups. Animals were randomly selected from each cage of group-housed animals, and a random component (referring to a random number on a pre-designed table) was used in order to assign the animals to their specific groups and/or treatment allocations. The order of animals being submitted to the behavioral tasks was arranged so that animals from different groups were being tested either at the same time or consecutively. One experimenter, who was not blinded, was assigned the task of administering treatments and distributing animals throughout an experiment. All observers scoring behavior and performing data analysis were blinded to treatment allocation.

#### Writhing test

The writhing test was performed as previously described [19]. Writhing behavior was induced by i.p. injection of 0.4 ml of acetic acid (0.6%) diluted in distilled water. Four groups of 6 mice each were pre-treated with i.p. injection of saline or HESc (3, 10 or 30 mg/kg) 30 min before acetic acid administration. Immediately after acetic acid injections, mice were placed in individual glass tanks and the number of cumulative writhing behaviors was measured over 15 min.

#### Formalin test

The formalin test was performed as previously described [20]. Five groups of 6 rats each were treated with i.p. injection of saline (Sal) or HESc (3, 10 or 30 mg/kg) 30 min before the administration of saline (Sal-Sal group) or 20 μl of 0.2% formalin (Sal-, HESc3-, HESc10- and HESc30-Formalin groups) on the plantar surface of the right hind paw. After formalin injection, rats were immediately placed in an open-field arena for the evaluation of nociceptive behavior according to the following score: 0, normal posture; 1, injected paw on the ground but not supporting the animal; 2, injected paw clearly raised; 3, injected paw being licked, nibbled, or shaken. The different parameters were timed every minute during the first 5 min and every 5 min afterwards in a 50 min session. The nociception index was determined by the mean of the pain score for each group.

#### Hot plate test

The hot plate test was performed as previously described [21]. The thermal stimulation of the hot-plate apparatus (10 cm wide glass cylinder on a hot plate - IITC Life Science Inc. CA, USA) was maintained at 55 **±** 1 °C. Mice were placed on the heated surface and the latency to jump or licking response was measured. Two control latencies at least 10 min apart were determined for each mouse. Animals were removed from the apparatus right after the expression of nociceptive behavior. A latency period of 30s was defined as complete antinociception (cut-off). Four groups of 5 mice each were treated with i.p. injection of saline or HESc (3, 10 or 30 mg/kg) 30 min before being placed in the apparatus and the thermal latency was evaluated every 30 min during 240 min.

#### Hypernociception test

The test consisted in evoking a hind paw flexion reflex with a hand-held force transducer adapted with a 0.7-mm^2^ polypropylene tip (Electronic von Frey; IITC Life Science, Woodland Hills, CA). The investigator was trained to apply the tip perpendicular to the central area of the hindpaw with a gradual increase in pressure. The end-point was characterized by the removal of the paw followed by clear flinching movements. After paw withdrawal, the intensity of the pressure was automatically recorded, and the final value for the response was obtained by averaging three measurements (basal mechanical nociceptive threshold). After collection of basal measures, 5 groups of 5 rats each were treated with i.p. injection of saline (Sal) or HESc (3, 10 or 30 mg/kg) 150 min after the administration of saline (Sal-Sal group) or prostaglandin E_2_ (PGE_2_, 100 ng/paw; Sal-, HESc3-, HESc10- and HESc30-PGE_2_ groups) in the plantar surface of right hind paw. After Sal or HESc injection, rats were immediately placed in acrylic cages (12 × 20 × 17 cm) with wire grid floors for evaluation of nociceptive behavior. The mechanical nociceptive threshold was evaluated by crescent pressure in the rat paw 30, 60 and 90 min after Sal or HESc treatment. Hypernociception was calculated as the difference between basal and post-treatment mechanical nociceptive thresholds (Δ).

#### Rota-rod test

To rule out possible nonspecific muscle-relaxant or sedative effects of HESc, mice were tested in the rota-rod apparatus, as previously described [21]. Twenty-four hours before the test session, all animals were observed in the rota-rod apparatus. Only mice that remained in the bar for 2 consecutive periods of 60s were considered for the test on the following day. Five groups of 6 mice each were treated with i.p. injection of saline, HESc (3, 10 or 30 mg/kg), or 2 mg/kg diazepam. Results are expressed as the time that the animals remained on the rota-rod in a 60s session in 4 different time-points: before treatment (basal) and 30, 60 and 90 min after the injections.

### Statistical analysis

Sample sizes were calculated using the freely downloadable software G Power for sample size calculation and performing an a priori analysis. The sample size was calculated using the power analyses for analyses of variance for fixed effects, using a one-factorial design (Experiment 1) or a multi-factorial design (Experiments 2 to 5). We have assumed a significance level (alpha) of 5% for all experiments, and power (probability of finding an effect) of 80%, using a two tailed test, with no expected attrition. Before conducting the statistical analysis, all variables were checked for normality (Shapiro–Wilk test) and homogeneity (Levene’s test), which validated the use of the parametric tests. Grouped data were analysed by one- or two-way analysis of variance (ANOVA) with repeated measures when appropriate followed by Bonferroni post hoc tests. For conditions in which repeated measures ANOVA was used, all variables were checked for and passed sphericity test. Using a two-way ANOVA allowed for the evaluation of a possible interaction effect between two given variables (variables described in the Results description of each Experiment). Whenever an interaction effect was observed, that effects was described in the results section. All graphical data presentations were created using Prism 5 (GraphPad Software), and all statistical tests were performed using PASW Statistics 18 (SPSS Statistics Software). A *p* value less than 0.05 was considered as a statistically significant difference.

## Results

### Writhing test

**Fig. 1:**
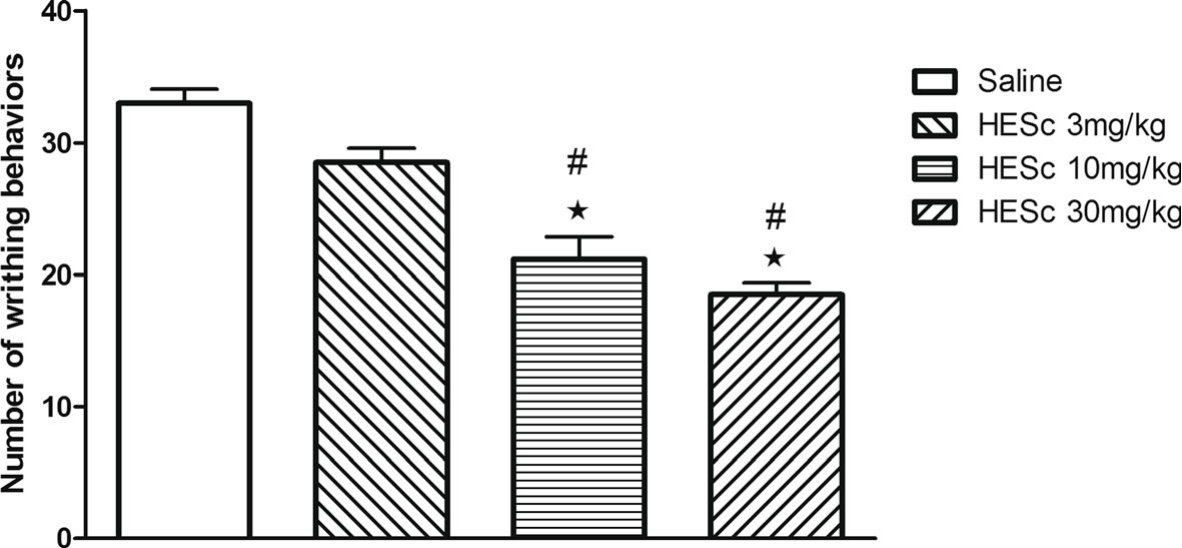
Effect of previous administration of saline or different doses of HESc (3, 10 or 30 mg/kg) on writhing behavior induced by intraperitoneal administration of 0.4 ml of acetic acid (0.6%) in mice (*n* = 6 per group). Data are presented as mean ± SEM. #*p* < 0.05 compared to Saline group; ★*p* < 0.05compared to HESc 3 mg/kg group. One-way ANOVA followed by Bonferroni test

One-way ANOVA indicated a significant difference between groups [F(3,20) = 29.88, *p* < 0.0001] (*n* = 6 per group, 4 groups). Bonferroni *post-hoc* test indicated that the group treated with 3 mg/kg HESc showed no significant difference when compared to the saline control group. However, higher doses of HESc (10 or 30 mg/kg) were significantly effective in reducing the number of writhes compared to both control and HESc 3 mg/kg groups (Fig. 1).

### Formalin test

**Fig. 2:**
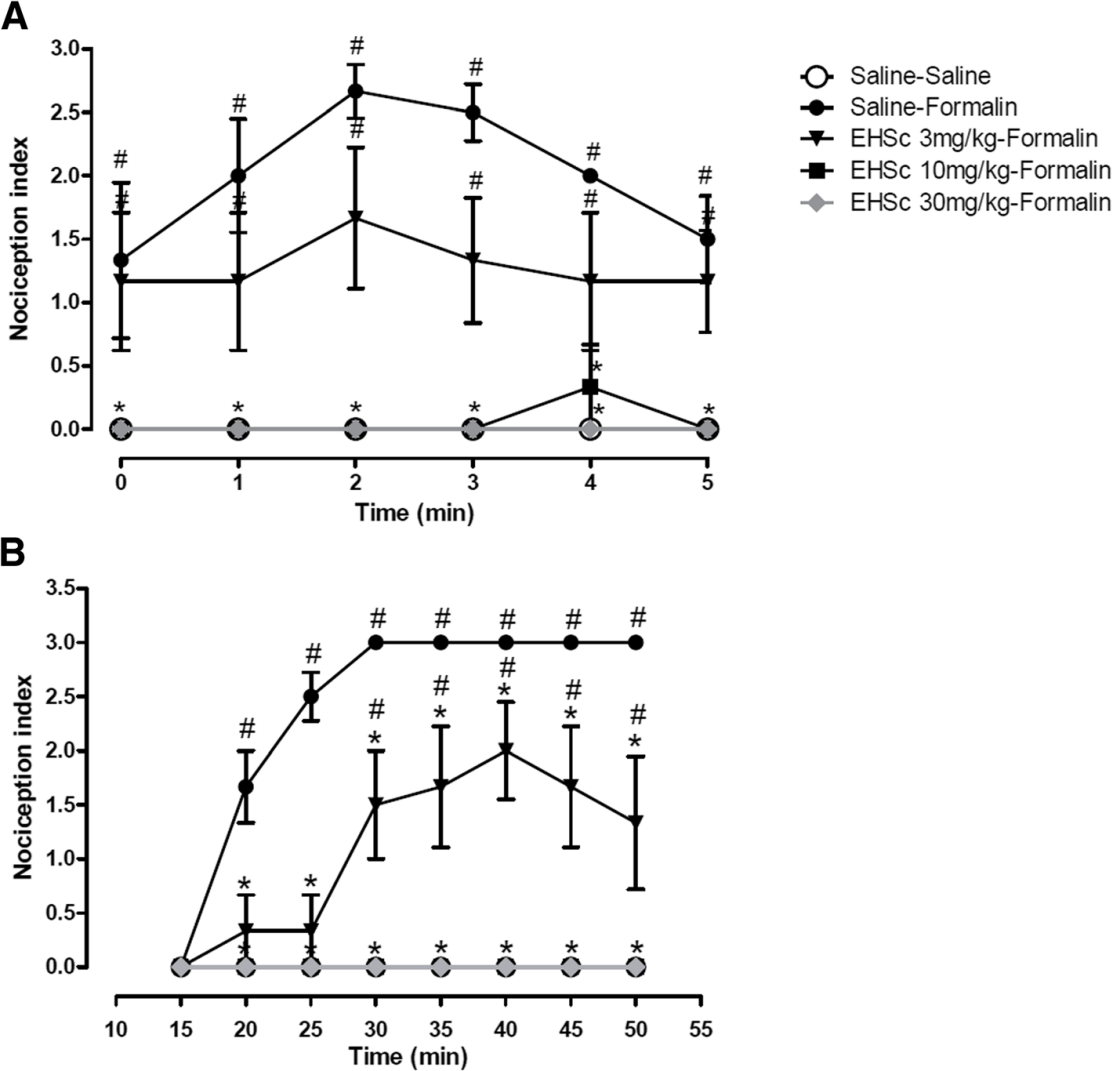
Effect of previous administration of saline or different doses of HESc (3, 10 or 30 mg/kg) on the acute (**a**) and inflammatory (**b**) phases of formalin-induced nociception (20 μl of 0.2% formalin injection on the plantar surface of right hind paw) in rats (*n* = 6 per group). Data are presented as mean ± SEM. #*p* < 0.05 compared to Saline-Saline group; *p < 0.05 compared to Saline-Formalin group. Two-way repeated measures ANOVA followed by Bonferroni test

Fig. 2 represents the effects of intraperitoneal administration of HESc (3, 10 or 30 mg/kg) on the acute (0-5 min, Fig. 2a) or inflammatory (15-50 min, Fig. 2b) phases of the formalin test session. Factors compared in the two-way repeated measures ANOVA were time (minutes 0–5 or 15–50), pre-treatment condition (saline vs HESc) and their interaction. In the acute phase, two-way repeated measures ANOVA indicated a significant effect of pre-treatment condition (Sal vs HESc3, HESc10 or HESc30) [F (4,125) = 21.88; *p* < 0.0001] (*n* = 6 per group, 5 groups). As expected, Bonferroni post hoc test indicated that formalin administration (Sal-Formalin group) enhanced nociception indexes at all time-points compared to Sal-Sal group, an effect was significantly reduced by previous administration of 3 mg/kg HESc. Moreover, 10 and 30 mg/kg HESc decreased nociception indexes during the entire acute phase of the test compared to Sal-Formalin group, showing the effect of the extract on acute nociception (Fig. 2a).

During the inflammatory phase, two-way repeated measures ANOVA indicated a significant interaction between time and pre-treatment factors [F (28,175) = 8.76; *p* < 0.0001]. Bonferroni post hoc test indicated that all doses of HESc were effective in reducing, either attenuating (3 mg/kg) or abolishing (10 and 30 mg/kg), formalin-induced enhanced nociception index (Fig. 2b).

### Hot plate test

**Fig. 3:**
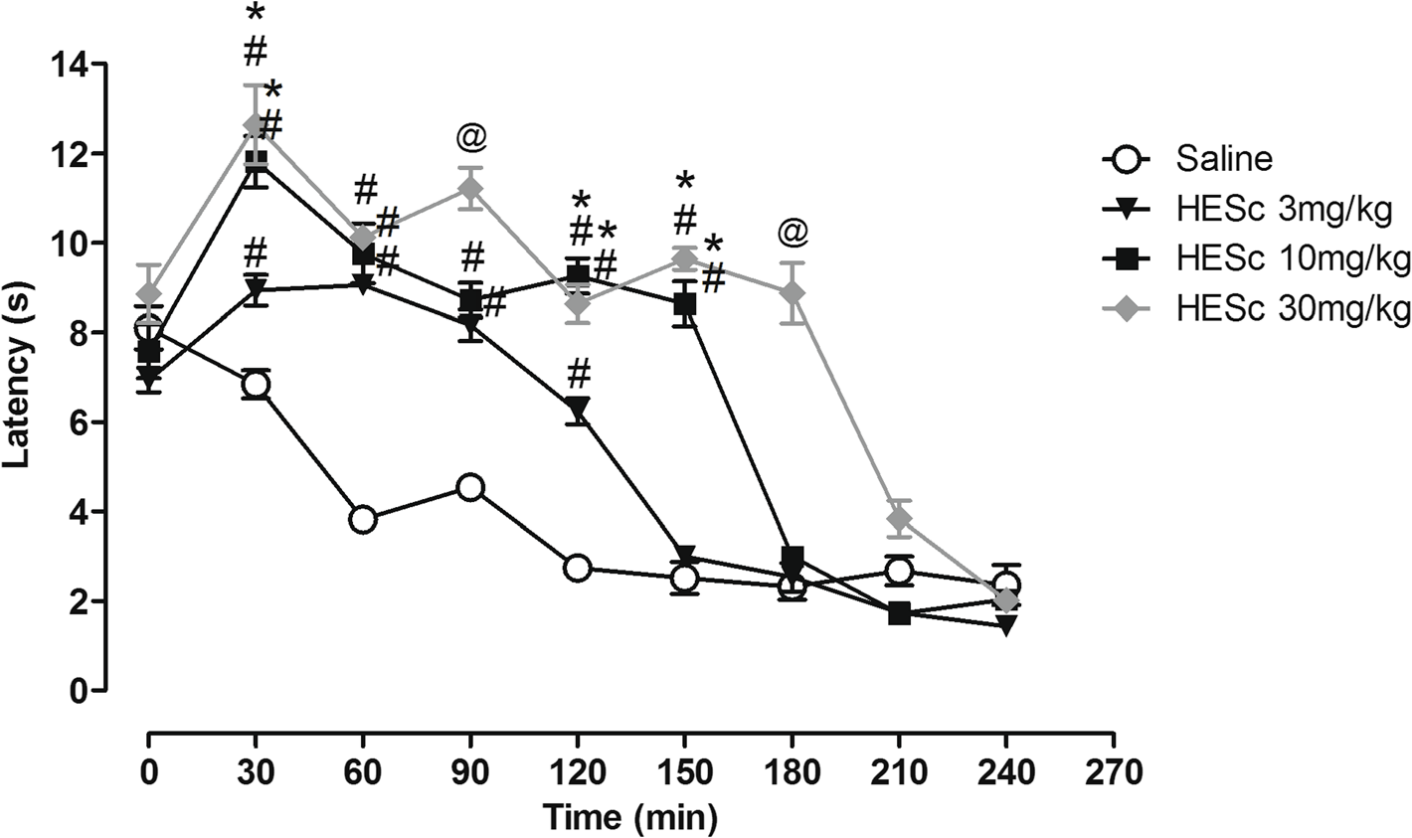
Antinociceptive effect of saline or HESc (3, 10 or 30 mg/kg) in the latency (seconds) to response to thermal stimulus in the hot plate test across time after treatment in mice (*n* = 5 per group). Data are presented as mean ± SEM. #*p* < 0.05 compared to Saline group in the same time-point; *p < 0.05 compared to HESc 3 mg/kg group in the same time-point; @*p* < 0.05 compared to all other groups in the same time-point. Two-way repeated measures ANOVA followed by Bonferroni test

Factors used in the two-way repeated measures ANOVA were time (minutes 0–240), pre-treatment condition (saline vs HESc) and their interaction. Two-way repeated measures ANOVA indicated a significant interaction effect between time and pre-treatment (Sal vs HESc3, HESc10 or HESc30) factors [F(24,128) = 19.50; *p* = 0.0001] (*n* = 5 per group, 4 groups). HESc pre-treatment significantly enhanced the latency to response to thermal stimulus compared to control (Sal) condition, effect that was dissipated across time (Fig. 3). Bonferroni post hoc test indicated that the group treated with 3 mg/kg HESc showed a significant increase in latency from minutes 30 to 120 (measures taken every 30 min), with no significant effect in the following time-points. For the dose of 10 mg/kg HESc, the time effect was extended to up to 150 min, reaching 180 min for the dose of 30 mg/kg HESc. Importantly, HESc at the doses of 10 and 30 mg/kg had a higher effect compared to the dose of 3 mg/kg on minute 30. Also, 30 mg/kg HESc led to a higher latency to response to thermal stimulus on time 90 compared to all other groups. Thus, with increasing dose there was not only a more pronounced effect, but also a dose-dependent effectiveness of HESc across time.

### Hypernociception test

**Fig. 4:**
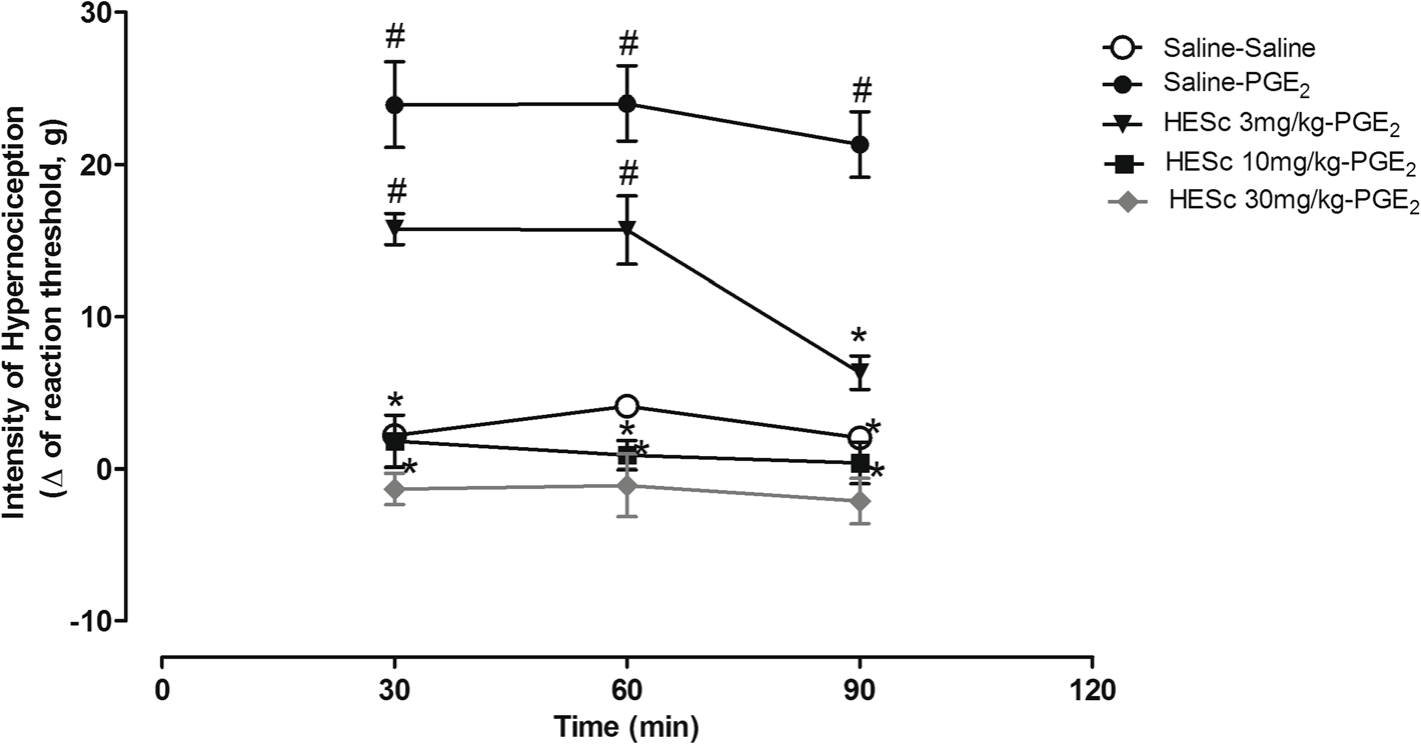
Effect of treatment with saline or HESc (3, 10 or 30 mg/kg) on the mechanic hypernociception induced by previous intraplantar injection of PGE_2_ in rats (*n* = 5 per group). Data are presented as mean ± SEM. #*p* < 0.05 compared to Saline-Saline group; *p < 0.05 compared to Saline-PGE_2_ group. Two-way repeated measures ANOVA followed by Bonferroni test

Factors used in the two-way repeated measures ANOVA were time (minutes 30–90), pre-treatment condition (saline vs HESc) and their interaction. Two-way repeated measures ANOVA showed a significant interaction effect between time and pre-treatment (Sal vs HESc3, HESc10 or HESc30) factors [F (8,38) = 3.66; *p* = 0.003] (*n* = 5 per group, 5 groups). Bonferroni post hoc test indicated that PGE_2_ treatment induced a higher difference between basal and post-treatment mechanical nociceptive thresholds compared to the Sal-Sal group throughout the session (Fig. 4). Pre-treatment with HESc was effective in inhibiting PGE_2_-induced hypernociception, with 10 and 30 mg/kg HESc being effective at all time-points, while the dose of 3 mg/kg was only effective at time 90 min.

### Rota rod test

Factors used in the two-way repeated measures ANOVA were time (minutes Basal – 90), treatment condition (saline vs HESc vs diazepam) and their interaction. Two-way repeated measures ANOVA indicated a significant interaction effect between time and treatment (Sal vs HESc3, HESc10 or HESc30) factors [F (12,150) = 3.02; *p* = 0.0008] (*n* = 6 per group, 5 groups). Bonferroni post hoc test indicated that animals treated with 2 mg/kg diazepam (benzodiazepine, BDZ) showed a significant reduction in the time spent on the device at 30 and 60 min after treatment, indicating sedative effect with impaired balance. Groups treated with different doses of HESc, on the other hand, did not differ from the Sal group, showing a lack of sedative effect or impairment in motor functions.

## Discussion

In the present study, the hydroalcoholic extract of the aerial parts of *Solidago chilensis* Meyen, formulation most commonly used for therapeutic properties, was tested in different models of pain and nociception in rodents. HESc exerted antinociceptive effects in models of inflammatory pain, as expected for an extract with proven anti-inflammatory action [4]. HESc also had antinociceptive action in models of non-inflammatory pain, such as nociceptive response to heat or a mechanical stimulus. Importantly, HESc did not alter time spent in the rota-rod apparatus. Thus, our data suggest that, when systemically administered, HESc decreases nociception without inducing a sedative effect.

The antinociceptive investigation in our study was initiated with the writhing test. This is an unspecific, although very sensitive, model for the evaluation of the antinociceptive properties of drugs in rodents, with a high predictive validity [20]. Our results showed a dose-dependent inhibition of acetic acid-induced writhing behavior by *S. chilensis* extract (Fig. 1). The nociceptive response in the writhing test has been shown to involve the release of endogenous mediators that stimulate nociceptive terminals, such as bradykinin, serotonin, histamine and prostaglandins [22]. Also, the nociceptive activity of acetic acid in this model requires the release of TNF-α and cytokines from local macrophages and mast cells [23]. In this scenario, the effects observed with HESc in the writhing test may have been mediated by direct or indirect inhibition of pro-inflammatory mediators or even by central modulation of the nociceptive transmission.

The antinociceptive action of HESc on the inflammatory phase of the formalin test (Fig. 2b) was an expected phenomenon. Tamura et al. [4] have demonstrated a significant effect of the extract on the mouse ear oedema induced by *Euphorbia milii* extract. This effect was associated with the mobilization of leukocytes to the inflammatory site in the air-pouch model, with significant anti-inflammatory effects. Tamura and colleagues [4] also demonstrated the presence of caffeoylquinic and di-caffeoylquinic acids as major constituents of this extract. Caffeoylquinic acids decrease the release of histamine from mast cells in vitro [24]. Importantly, Parada et al. [25] showed that a pretreatment with cromolyn sodium (mast cell stabilizer) in the formalin model significantly reduced the inflammatory phase of pain. Thus, HESc-induced inhibition in the inflammatory phase of the formalin model may have been promoted by a reduction in histamine release from mast cells.

Interestingly, HESc also induced a significant reduction in the non-inflammatory nociceptive phase of pain in the formalin model (Fig. 2a). Formalin injection has been shown to cause a direct chemical stimulation in the spontaneous activity of nociceptive type C fibers [26]. Terminals of C-fibers are one of the targets of brain circuits of the pain modulatory system [27]. Thus, the effects of HESc on the acute (nociceptive) phase of the formalin test are most likely related to a direct action on nociceptive terminals, probably due to the release of excitatory aminoacids, nitric oxide and substance P. Therefore, we raised the hypothesis that further compounds in the extract (not yet identified) might have significant effects on the central transmission of pain, which takes place in this initial (non-inflammatory) phase of the formalin test.

To test this hypothesis, we evaluated the effects of HESc treatment on mice’s thermal response in the hot plate test. This test is characterized by leading to rapid responses to noxious stimuli mediated by the activation of nociceptors, which conduct the impulse to the dorsal horn and then to cortical centers [28]. C fibers are stimulated by the activation of vanilloid receptor (VR-1), responsible for detecting external temperature rise [29]. Thus, if HESc-induced antinociceptive effects were mediated by central systems related to the expression of pain, this extract would also exert antinociceptive effects in the hot plate test. We found that HESc at all doses significantly increased resistance time in this test. This effect was more pronounced and lasted longer with an increase in dose (Fig. 3). Therefore, the antinociceptive effect of HESc in the hot plate test indicates a possible action of HESc (or its compounds) in higher spinal regions.

To evaluate the effect of the extract of *S. chilensis* on hypernociception, we used the model of the electronic Von Frey. The administration of PGE_2_ alone induces a high sensitivity in rodents, with low threshold to pain, as shown in our control group (PGE_2_-Sal vs Sal-Sal groups, Fig. 4). Treatment with HESc led to a dose-dependent reduction in hypernociception and increase in pain threshold, with hypernociception being abolished by the highest dose. PGE_2_ is synthesized in inflammatory sites, acting as a potent vasodilator and inducing the production of several chemo-attractors, such as leukocytes and pro-inflammatory cytokines [30]. These mediators also act in acute pain sensitizing nerve terminals of types Aδ and C nociceptive fibers through EP1 receptors [31]. Thus, the anti-inflammatory mediators present in the extract of *S. chilensis* would interfere with the process in order to reduce the migration of leukocytes to the site of inflammation, as reported by Tamura et al. [4]. This effect would also be associated with the possible direct action of HESc on central nociceptive terminals.

**Fig. 5:**
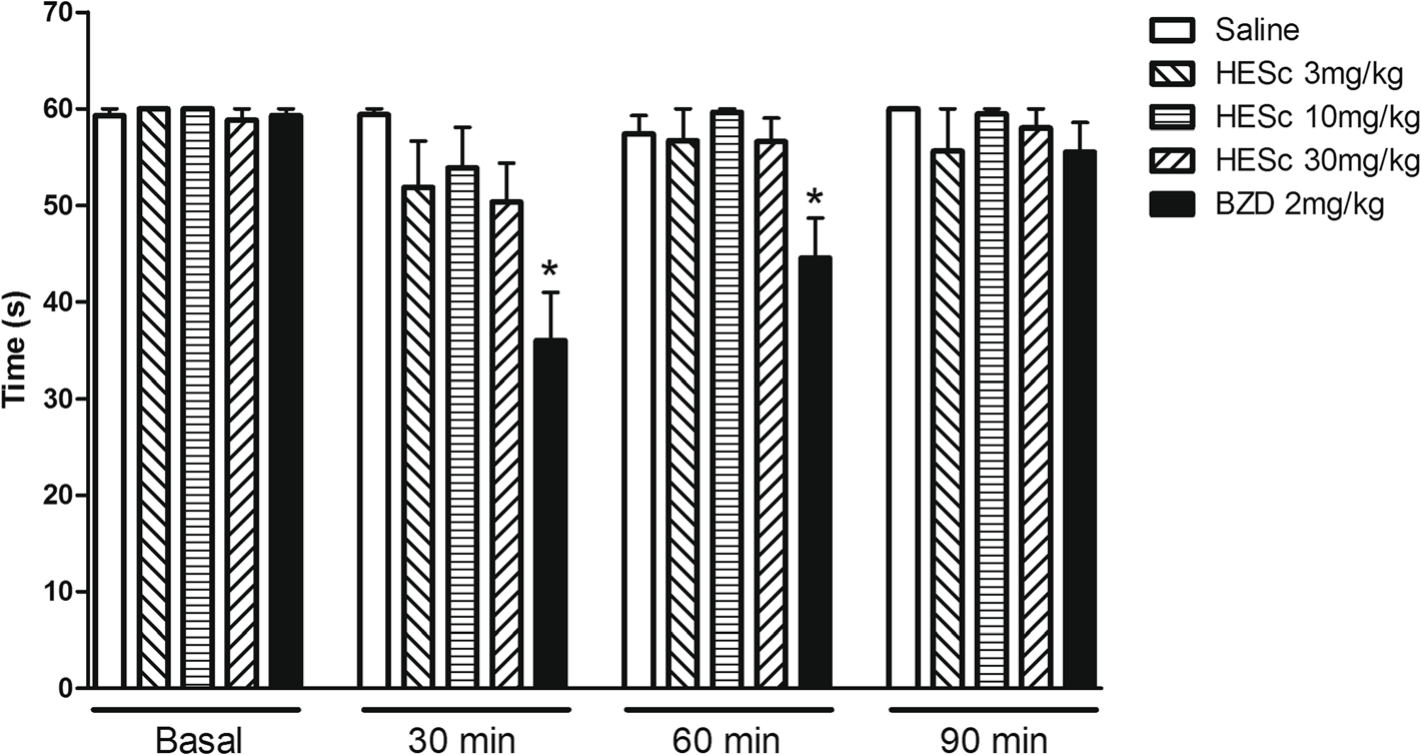
Effect of treatment with saline, HESc (3, 10 or 30 mg/kg) or diazepam (BDZ, 2 mg/kg) on the motor coordination of mice (*n* = 6 per group) in the rota rod test. Data are presented as the mean ± SEM. *p < 0.05 compared to Saline group. Two-way repeated measures ANOVA followed by Bonferroni test

Together, our results corroborate previous preliminary findings in humans indicating a possible antinociceptive action of *S. chilensis* [16]. Of note, the doses of HESc used in the present study did not affect motor function in rodents (Fig. 5), excluding a possible bias of sedative effects in our study. A previous study from our group also demonstrated that at the highest dose tested in the present study (30 mg/kg), no deaths and no adverse effects were observed in mice following several acute toxicity tests [32]. More important, our findings show that HESc exerts antinociceptive effects in both inflammatory and non-inflammatory models of nociception and pain. This suggests a broader action of *S. chilensis* in pain management than those specifically related to inflammatory mechanisms. It is important to note, however, that no positive controls were included in the present study, limiting the comparison between the antinociceptive effects of HESc and other standard drugs. Thus, further studies should address specific compounds of this extract that might have particular pain-related actions, possibly indicating new perspectives to clinical practice.

## Conclusions

Our data suggest that, when systemically administrated, HESc decreases nociception without inducing a sedative effect. Importantly, this effect was observed in both inflammatory and non-inflammatory models of pain and nociception, suggesting a specific non-inflammatory mechanism of HESc on pain. Our findings indicate that *S. chilensis* might be an important adjuvant in pain management.

## Abbreviations

BDZ: Benzodiazepine = diazepam
EP1 receptors: Prostaglandin E receptor 1
HESc: hydroalcoholic extract of *Solidago chilensis*
HESc10: Hydroalcoholic extract of *Solidago chilensis* 10 mg/kg
HESc3: Hydroalcoholic extract of *Solidago chilensis* 3 mg/kg
HESc30: Hydroalcoholic extract of *Solidago chilensis* 30 mg/kg
IL-1: Interleukin 1
PGE2: Prostaglandin E2
SUS: Brazilian public health program
VR-1: vanilloid receptor 1

## References

[1] Souza DMF, Sá RD, Araújo EL, Randau KP. Anatomical, phytochemical and histochemical study of Solidago chilensis Meyen. An Acad Bras Cienc. 2018;90(2 suppl 1):2107-20. 10.1590/0001-3765201720160280 28678961

[2] Goulart S, Moritz MI, Lang KL, Liz R, Schenkel EP, Fröde TS (2007). Anti-inflammatory evaluation of Solidago chilensis Meyen in a murine model of pleurisy. J Ethnopharmacol.

[3] Liz R, Vigil SV, Goulart S, Moritz MI, Schenkel EP, Fröde TS (2008). The anti-inflammatory modulatory role of Solidago chilensis Meyen in the murine model of the air pouch. J Pharm Pharmacol.

[4] Tamura EK, Jimenez RS, Waismam K, Gobbo-Neto L, Lopes NP, Malpezzi-Marinho EA, Marinho EA, Farsky SH (2009). Inhibitory effects of Solidago chilensis Meyen hydroalcoholic extract on acute inflammation. J Ethnopharmacol.

[5] Jorge SSA. Plantas Medicinais: Coletânea de Saberes. 2009. http://www.agronomiaufs.com.br/index.php/download-e-videos/category/75-downloads.

[6] Posse JC. Medicinal plants used by SUS users in Paquetá and Santa Teresa neighborhoods. An ethnobotany approach. Rio de Janeiro. Dissertation. Post-graduate program in pharmaceutical sciences, Faculty of Pharmacy, Federal University of Rio de Janeiro, Rio de Janeiro (in Portuguese). 2007, 115p.

[7] Amorim CC (2009). Ethnobotany of land medicine in the Coxilha Rica region, SC. Rev Bras Agroecologia.

[8] Calábria L, Cuba GT, Hwang SM, Marra JCF, Mendonça MF, Nascimento RC, Oliveira MR, Porto JPM, Santos DF, Silva BL, Soares TF, Xavier EM, Damasceno AA, Milani JF, Rezende CHA, Barbosa AAA, Canabrava HAN (2008). Ethnobotanical and ethnopharmacological survey of medicinal plants in Indianopolis, Minas Gerais, Brazil. Revista Brasileira de Plantas Medicinais.

[9] Kffuri CW. Ethnobotany of medicinal plants in Senador Firmino (Minas Gerais State). Dissertation, Federal University of Viçosa, 2008;88p.

[10] Oliveira HB, Kffuri CW, Casali VWD (2010). Ethnopharmacological study of medicinal plants used in Rosário da Limeira, Minas Gerais, Brazil. Rev Bras Farmacogn.

[11] Vila R, Mundina M, Tomi F, Furlán R, Zacchino S, Casanova J, Cañigueral S (2002). Composition and antifungal activity of the essential oil of Solidago chilensis. Planta Med.

[12] Duarte MC, Figueira GM, Sartoratto A, Rehder VL, Delarmelina C (2005). Anti-Candida activity of Brazilian medicinal plants. J Ethnopharmacol.

[13] Bucciarelli A, Minetti A, Milczakowskyg C, Skliar M (2010). Evaluation of gastroprotective activity and acute toxicity of Solidago chilensis Meyen (Asteraceae). Pharm Biol.

[14] de Barros M, Mota da Silva L, Boeing T, Somensi LB, Cury BJ, de Moura Burci L, Santin JR, de Andrade SF, Monache FD, Cechinel-Filho V (2016). Pharmacological reports about gastroprotective effects of methanolic extract from leaves of Solidago chilensis (Brazilian arnica) and its components quercitrin and afzelin in rodents. Naunyn Schmiedeberg's Arch Pharmacol.

[15] Melo AM, Bittencourt P, Nakutis FS, Silva AP, Cursino J, Santos GA, Ashino NG, Velloso LA, Torsoni AS, Torsoni MA (2011). Solidago chilensis Meyen hydroalcoholic extract reduces JNK/IκB pathway activation and ameliorates insulin resistance in diet-induced obesity mice. Exp Biol Med (Maywood).

[16] da Silva AG, de Sousa CP, Koehler J, Fontana J, Christo AG, Guedes-Bruni RR (2010). Evaluation of an extract of Brazilian arnica (Solidago chilensis Meyen, Asteraceae) in treating lumbago. Phytother Res.

[17] da Silva AG, Machado ER, de Almeida LM, Nunes RM, Giesbrecht PC, Costa RM, Costa HB, Romão W, Kuster RM (2015). A clinical trial with Brazilian Arnica (Solidago chilensis Meyen) glycolic extract in the treatment of tendonitis of flexor and extensor tendons of wrist and hand. Phytother Res.

[18] Gobbo-Neto L, Lopes NP (2008). On line identification of chlorogenic acids, sesquiterpene lactones, and flavonoids in the Brazilian arnica Lychnophora ericoides Mart. (Asteraceae) leaves by HPLC–DAD–MS and HPLC–DAD–MS/MS and a validated HPLC–DAD method for their simultaneous analysis. J Agric Food Chem.

[19] Niemegeers CJ, Van Bruggen JA, Janssen PA (1975). Suprofen, a potent antagonist of acetic acid-induced writhing in rats. Arzneimittelforschung..

[20] Le Bars D, Gozariu M, Cadden SW (2001). Animal models of nociception. Pharmacol Rev.

[21] Vaz ZR, Filho VC, Yunes RA, Calixto JB (1996). Antinociceptive action of 2-(4-bromobenzoyl)-3-methyl-4,6-dimethoxy benzofuran, a novel xanthoxyline derivative on chemical and thermal models of nociception in mice. J Pharmacol Exp Ther.

[22] Whittle BA (1964). Release of a Kinin by intraperitoneal injection of chemical agents in mice. Int J Neuropharmacol.

[23] Ribeiro RA, Vale ML, Thomazzi SM, Paschoalato AB, Poole S, Ferreira SH, Cunha FQ (2000). Involvement of resident macrophages and mast cells in the writhing nociceptive response induced by zymosan and acetic acid in mice. Eur J Pharmacol.

[24] Kimura Y, Okuda H, Okuda T, Hatano T, Agata I, Arichi S (1985). Studies on the activities of tannins and related compounds from medicinal plants and drugs. VI. Inhibitory effects of caffeoylquinic acids on histamine release from rat peritoneal mast cells. Chem Pharm Bull (Tokyo).

[25] Parada CA, Tambeli CH, Cunha FQ, Ferreira SH (2001). The major role of peripheral release of histamine and 5-hydroxytryptamine in formalin-induced nociception. Neuroscience..

[26] Hunskaar S, Fasmer OB, Hole K (1985). Acetylsalicylic acid, paracetamol and morphine inhibit behavioral responses to intrathecally administered substance P or capsaicin. Life Sci.

[27] Snider WD, McMahon SB (1998). Tackling pain at the source: new ideas about nociceptors. Neuron..

[28] Dickenson AJ, Besson JM (1997). The pharmacology of pain.

[29] Julius D, Basbaum AI (2001). Molecular mechanisms of nociception. Nature.

[30] Kaur S, Kaur H, Singh PP (2004). Induction of colony-stimulating factors by a 30-kDa secretory protein of mycobacterium tuberculosis H37Rv. Eur Cytokine Netw.

[31] Rady JJ, Campbell WB, Fujimoto JM (2001). Antianalgesic action of nociceptin originating in the brain is mediated by spinal prostaglandin E(2) in mice. J Pharmacol Exp Ther.

[32] Paula-Freire LIG, Malpezzi-Marinho ELA, Molska GR, Kohn DO, Correa L, Marinho EAV (2014). Evaluation of the acute toxicity of the hydroalcoholic extract of solidago chilensis meyen (arnica do campo) in mice. Am J Phytomedicine Clin Therapeutics.

